# Health Tract, No. 164

**Published:** 1863-08

**Authors:** 


### SICKNESS NOT CAUSELESS.

There never can be disease without a cause; and almost always the cause is in the person who is ill; he has either done something which he ought not to have done, or he has omitted something which he should have attended to.

Another important item is, that sickness does not, as a general thing, come on suddenly; as seldom does it thus come, as a house becomes enveloped in flames, on the instant of the fire first breaking out. There is generally a spark, a tiny flame, a trifling blaze. It is so with disease, and promptitude is always an important element of safety and deliverance. A little child wakes up in the night with a disturbing cough, but which, after a while passes off, and the parents feel relieved; the second night the cough is more decided; the third, it is croup, and in a few hours more, the darling is dead!

Had that child been kept warm in bed the whole of the day after the first coughing was noticed, had fed lightly, and got abundant, warm sleep, it would have had no cough the second night, and the day after would have been well.

An incalculable amount of human suffering, and many lives would be saved every year, if two things were done uniformly. First, when any uncomfortable feeling is noticed, begin at once, trace the cause of it and avoid that cause ever after. Second, use means at once to remove the symptom; and among these, the best, those which are most universally available and applicable, are rest, warmth, abstinence, a clean person, and a pure air. When animals are ill, they follow nature's instinct, and lie down to rest. Many a valuable life has been lost by the unwise efforts of the patient to "keep up," when the most fitting place was a warm bed and a quiet apartment.

Some persons attempt to "harden their constitutions," by exposing themselves to the causes which induced their sufferings, as if they could by so doing, get accustomed to the exposure, and ever thereafter endure it with impunity. A good constitution, like a good garment, lasts the longer by its being taken care of. If a finger has been burned by putting it in the fire, and is cured never so well, it will be burned again as often as it is put in the fire; such a result is inevitable. There is no such thing as hardening one's self against the causes of disease. What gives a man a cold to-day, will give him a cold to-morrow, and the next day, and the next. What lies in the stomach like a heavy weight to-day, will do the same to-morrow; not in a less degree, but a greater; and as we get older, or get more under the influence of disease, lesser causes have greater ill effects; so that the older we get, the greater need is there for increased efforts to favor ourselves, to avoid hardships and exposures, and be more prompt in rectifying any "symptom," by rest, warmth, and abstinence.

